# S12-5 Physical activity trends among second-level Active School Flag programme 2019-2021

**DOI:** 10.1093/eurpub/ckad133.062

**Published:** 2023-09-11

**Authors:** Kwok Ng, Caera Grady, Fiona McHale, Kathleen McNally, Maeve Conneelly, Catherine Woods

**Affiliations:** Physical Activity for Health Research Cluster, Department of Physical Education and Sport Sciences, University of Limerick, Limerick, Ireland; Faculty of Education, University of Turku, Rauma, Finland; School of Educational Sciences and Psychology, Uni; Physical Activity for Health Research Cluster, Department of Physical Education and Sport Sciences, University of Limerick, Limerick, Ireland; Physical Activity for Health Research Cluster, Department of Physical Education and Sport Sciences, University of Limerick, Limerick, Ireland; Physical Activity for Health Research Cluster, Department of Physical Education and Sport Sciences, University of Limerick, Limerick, Ireland; Physical Activity for Health Research Cluster, Department of Physical Education and Sport Sciences, University of Limerick, Limerick, Ireland; Physical Activity for Health Research Cluster, Department of Physical Education and Sport Sciences, University of Limerick, Limerick, Ireland

## Abstract

**Purpose:**

Schools in the second level Active School Flag (ASF) programme are required to carry out a whole school survey to capture the student voice within the programme. Data from these surveys can be used to measure changes in physical activity behaviours at the school level. This presentation outlines the changes in physical activity levels over a three-year period from students in ASF schools.

**Methods:**

From five schools involved in the ASF programme from 2019 to 2021, students reported frequencies of 60 minutes of aerobic physical activity, muscle strengthening, and active transport. Students were grouped into inactive (0-2 days), somewhat active (3-4 days), active (5-6 days) and daily active (7 days), as well as meeting muscle strengthening guidelines (3+ days/week) and not meeting (0 – 2 days). Furthermore, only students who lived within 5km of their school were included in the active transport analyses, where active transport was a combination of walking and cycling. Contingency tables were produced, and logistic regressions were used to analyse changes between 2019 (reference), 2020 (COVID-19 lockdown), and 2021, with age as confounders and stratified by gender.

**Results:**

There was an 80% response rate across the five schools. Of the 7646 students (male 33%, female 65%, other 2%), minimal changes in ‘daily active’ students were observed from 2019 (12%), 2020 (13%), to 2021 (12%), whereas ‘inactive’ students decreased from 25% in 2019 to 23% in both 2020 and 2021. Rates of meeting muscle-strengthening guidelines remained steady over the years for both males and females. Active transport increased from 55% in 2019 to 66% in 2021 for males and 50% in 2019, 63% in 2020, to 65% in 2021 for females. In addition to these overall trends, idiosyncratic analyses of the schools revealed different trends from being in the ASF programme.

**Conclusions:**

There were high level of responses from the whole school survey. The ASF intervention contains adaptable components due to the complex adaptive sub-systems and may shed light on the differences in physical activity over time. Furthermore, COVID-19 restrictions may have affected the program-related results.

